# Public Health, Hygiene and Bacteriology

**Published:** 1895-12

**Authors:** Ernest Wende

**Affiliations:** Health Commissioner of the city of Buffalo, N. Y


### PUBLIC HEALTH, HYGIENE AND BACTERIOLOGY.

Conducted by ERNEST WENDE, M. D.,
Health Commissioner of the City of Buffalo, N. Y.

### October Health in Buffalo.

By FRANKLIN C. GRAM, Registrar of Vital Statistics.

THERE was no material change in the city's death-rate for October from that of the previous month, although there was quite a difference between this and the same months of several years previous. Consumption claimed the greatest number of victims—namely, 34, as against 42 for the same month last year. Next comes pneumonia with 27, as against 20 last year. A synopsis of the principal communicable diseases in which physicians are interested is presented in the following table :

| | Tuberculosis. | Enteric Fever. | Diphtheria. | Diphtheritic Croup. | Membranous Croup. | Scarlet Fever. | Measles. | Pertussis. |
|---|---|---|---|---|---|---|---|---|
| Cases reported, Oct., 1895.. | 41 | 71 | 97 | 8 | 10 | 25 | 2 | 1 |
| Deaths, Oct., 1895......... | 34 | 20 | 23 | 1 | 9 | 3 | 0 | 1 |
| Cases reported, Oct., 1894.. | 0 | 80 | 63 | 1 | 5 | 65 | 4 | 2 |
| Deaths, Oct., 1894......... | 42 | 22 | 27 | 7 | 8 | 2 | 0 | 0 |
| Deaths, Oct., 1893 ......... | 41 | 13 | 16 | 3 | 9 | 9 | 1 | 5 |
| Deaths, Oct., 1892......... | 42 | 42 | 30 | 0 | 0 | 12 | 1 | 3 |
| Deaths, Oct., 1891......... | 41 | 39 | 19 | 0 | 0 | 11 | 1 | 2 |

Consumption was not reported in 1894. The apparent inaccuracy, in 1894, between the cases of diphtheritic and membranous croup reported and the number of deaths is accounted for in several ways. Some physicians, at that time, claimed to be unaware that these diseases should be reported to this department.

The deaths, and rate per thousand, for the month of October, for this and the past four years are as follows : 1895, deaths 394, rate 14.08 ; 1894, deaths 440, rate 16.76 ; 1893, deaths 480, rate 19.20 ; 1892, deaths 464, rate 19.53 ; 1891, deaths 451, rate 21.22. Although the population has gradually increased, yet the death-rate is steadily decreasing.

There were 716 births and 262 marriages during October, 1895. It is remarkable how the number of premature and still-births keep up, the former being 28 and the latter 33.

---

Armstrong (*Medical Record*) gives the following hints for the use of alcohol in fevers : 1. If the tongue becomes dry, discontinue ; if moister, continue. 2. If the pulse becomes quicker it does harm ; if slower, good. 3. If the skin becomes moister, good is being done. 4. If the breathing becomes easier continue its administration.

